# Infectious Endometritis

**Published:** 1903-03

**Authors:** J. W. Kenney

**Affiliations:** San Antonio, Texas


THE
### TEXAS MEDICAL JOURNAL.

ESTABLISHED JULY, 1885.

PUBLISHED MONTHLY.—SUBSCRIPTION $1.00 A YEAR.

VOL. XVIII. AUSTIN, MARCH, 1903. No. 9.

### Original Contributions.

For Texas Medical Journal.

### Infectious Endometritis.

BY J. W. KENNEDY, M. D., SAN ANTONIO, TEXAS.

Infectious endometritis, like many other diseases, is a decided reproach upon our civilization. That such is the case will scarcely meet with denial. It is unknown to the savage before being approached by the missionaries of civilized countries. With the advance of civilization and the betterment of our facilities for travel, we find this disease likewise progressive. In fact, so well has its etiological factor obeyed the command to increase and multiply that today it produces a large per cent. of the work alloted to the gynecologist. The numerous wrecked lives due directly to such infection place it in the front rank of all ailments now menacing the lives of women.

Being a preventable disease, it all the more loudly demands the attention not only of the medical profession, but of the law-making bodies as well. As it is the innocent that suffer most there should be some mode devised by which they can be protected. If such protection can not be gained by law, then by educating the women—the highest as well as the lowest, for the disease is no respecter of persons—so that they may understand not only the symptoms of the disease itself, but more important still, those of the infectious vaginitis that very often if not invariably proceeds it. It is only in the early recognition of these symptoms that she may hope to escape the horrible visitations such a disease is sure to bring. Until prophylactic measures become more general, however, the gynecologist will continue to find in his clientele an abundance of diseased uteri and adnexa uteri due directly to gonorrheal infection and indirectly to ignorance. To these he must devote his skill and attention.

Pathologically chronic infectious endometritis presents conditions similar to those produced by many other local disturbances of the nutrition of the uterus. Before the discovery of the specific cause of the disease it was considered in the light of a simple endometritis; this in turn embraced all diseases, whether inflammatory or not, except malignant growths. The microscope, together with a better understanding of the clinical history, has cleared away this nebulæ of confused conditions. Extensive pus and round cell infiltration characterize the acute stage. The endometrium becomes thickened and is covered with papillomatous growths. In the chronic stage we find the pus becoming more and more localized. This localization is usually in the neighborhood of the glands—the so-called pockets, which are in reality all that remains of pabulum producing endometrium. Consequently, these are the only points at which the gonococci can continue to propagate themselves. In this condition the uterus may remain indefinitely, the gonococci becoming less virulent by natural attenuation. This natural attenuation being aided in a measure at least by the supply of pabulum necessary for the gonococci to live upon becoming exhausted. These diplococci not only become imbedded in the intercellular spaces of gland epithelium, but likewise find their way into the sub-epithelial connective tissue. Finally there is produced a condition of pyometra, due, no doubt, to the formation of a pyogenic membrane over the entire inner surface of the uterus. This condition we recognize as the work of the streptococci and staphylococci—pyogenic bacteria that are usually associated with the gonococci. As the disease progresses we find this same condition gradually being implanted upon the uterine adnexa and parametrium. Glandular hyperplasia and hypertrophy take place, which finally results in the formation of pus and masses of necrotic tissue.

The clinical history of these cases is not always characteristic of the disease. So insidious is the invasion of the endometrium by the gonococci that at times the innocent or unknowing ones become extensively infected without realizing the serious nature of their trouble. Such instances are not uncommon, and they are usually found among the better class of married women whose husbands have had gonorrhea and been cured of it one or more times prior to marriage. In such husbands natural attenuation has rendered the gonococci less virulent, but when emplanted upon the healthy vaginal mucosa or endometrium this latent condition is gradually revived, and sooner or later the entire interior of the uterus is infected. From hence the gonococci, together with other pyogenic bacteria, find their way along the fallopian tubes to the peritoneal cavity. As before stated, it is in these unsuspecting ones that the disease becomes far advanced before a physician is consulted. Very often the patient is not even then informed as to the true nature of her disease. For obvious reasons such information is withheld. In the more pronounced cases we find a clinical picture that is pathognomonic of the disease. In such cases we have a definite history of acute infectious vaginitis, which, after a week or so, is followed by an inflammatory condition of the uterus. As the disease becomes chronic the discharge becomes less and the pain, tenderness and constitutional symptoms subside. This hegira of symptoms is only temporary, however. The purulent discharge, together with dull aching pains coming on ever and anon in the pelvic region, keeps the patient always fearful that the disease is still at work. This fear is converted into realism when the morbid process reaches the tubes and ovaries. A life of continued invalidism is now mapped out for the patient, and unless radical surgical measures are resorted to this map will be found a correct one. The patient goes from doctor to doctor. Local applications to the endometrium—medicated tampons, hot and astringent douches and the curette are used ostensibly for the relief of the symptoms, but in reality only lengthening the clinical history and disheartening the patient.

The cause of infectious endometritis was for many years a problem. Today but one factor is considered in the causation of this disease—the gonococcus of Neisser. The finding of this pathogenic parasite is all that is required to diagnose the disease. In chronic cases this is sometimes very difficult. The clinical history and an intimate acquaintance with the action of the gonococci upon the endometrium, however, seldom leave us in doubt as to the nature of the disease, even when a careful search for the diplococci has been in vain. An obstinate catarrh of the genital tract that refuses to yield to all the ordinary modes of treatment is, if not associated with struma, considered sufficient to warrant a diagnosis of infectious endometritis.

Infection of the endometrium, while serious enough in itself, is as nothing compared to the sequelæ which are brought about by an extension of the infection to the uterine adnexa and parametrium. It is these manifestations of latent gonorrhea that cause the patient to advance from the plane of pain, discomfort and misery to one of danger and perhaps death. This latter—death—from the meager history we have of such conditions, must have been the usual result prior to the middle of the nineteenth century.

The prognosis, when infectious endometritis has been definitely diagnosed, is always to be guarded—a possible or probable prophecy is more advisable than a positive declaration as to the outcome. In 1873 one of those highest in authority on the subject pronounced gonorrhea in the male an incurable disease. If such be true of infectious urethritis in the male, it is self-evident that the same condition in the female after the endometrium has become involved is a far more formidable disease. Regarding the latter affection, should radical surgical measures not be considered, the author of this paper would be willing to let his views go on record as agreeing with those of Dr. Noggerath. Since 1873, however, those great advances in surgery fathered by McDowell, Long and Lister have become more than ever firmly fixed realisms in scientific surgical practice. Today we are prepared to offer to women with infectious endometritis a favorable prognosis, provided they will submit to the necessary surgical treatment. We can at least hold out to them a prospect of relief from a disease which, in so far as medicine is concerned at the present time, is an impossibility. That a method of treatment specific in nature is needed none will deny. To my mind a cure for this disease is demanded as forcibly as is a cure for tuberculosis or cancer. Even more so, because the nature of the disease is so well understood and the suffering it entails is two-fold more than that of either of the above mentioned diseases. Local applications to the lining membrane of the uterus for the cure of the catarrhal condition due to gonorrheal infection have been used ever since the disease has been recognized. Few, if any, of these applications have been beneficial, and not one of them has resulted in a cure. Each application subjects the patient to discomforts such as are naturally incident to manipulating an inflamed uterus. Very often such manipulation sets up a violent metritis, and perhaps salpyngitis, that forces the patient to bed for a week or two. Local applications of any kind are never curative, seldom palliative, and often productive of serious consequences, and are therefore to be condemned as unsuitable in such cases.

Thorough curettment after the subsidence of any acute manifestation of the disease is a favorite mode of treatment. This usually, if not always, fails in the purpose for which it was intended. No matter how skillful the operator or how careful he may be, islands of diseased tissue will be left. From these the disease rapidly extends to its former proportions. A repetition of the curettment is often advised, and may at times meet with success. As a rule, however, it meets with the same result as did the first.

Atmocausis of late seemed to offer a simple means of curing these cases, as well as others, where the endometrium is diseased. Theoretically it would appear that the steam would have a uniform effect upon the diseased tissue, thus having an advantage over the curette in that there would be no diseased tissue left to serve as a focus for reinfection. Its adherents claimed for it that it is a safe and painless procedure. Individually, I have not found it efficient in infectious endometritis. My experience has, however, been limited to three cases. This is not enough to test any therapeutic measure. It would seem to be an ideal treatment for those cases where the diplococci had not entered the sub-epithelial connective tissue. Unfortunately the time necessary for this migration is not known. It is probably too short for one to hope to anticipate it by atmocausis. The fact that steaming the uterus practically destroys its cavity would not deter us in its use provided it cured the disease, for the disease has, in a majority of instances, rendered the patient sterile. It undoubtedly has one great advantage over the curette in that it is absolutely aseptic. With the curette there is always the possibility of adding to the seriousness of the situation by the introduction of infected matter from the outside, thereby producing a still more mixed infection.

The one certain cure for chronic infectious endometritis is to be found in vaginal hysterectomy, and it is to this procedure that the surgeon must eventually turn if he desires to rid his patient of this formidable and at the same time loathsome disease. The operation is a comparatively simple one at the present day—the danger to the patient not being one-tenth that which she permits herself to suffer if the infected uterus is allowed to remain until the adnexa become infected. This latter is certain to be the case sooner or later, and then a more serious operation is demanded or the patient's life must be sacrificed. On account of the popular belief among the laity of the seriousness of the operation some difficulty may be encountered in gaining the patient's consent to its performance. This difficulty will become less and less as the good results of the operation are witnessed by womankind. The greatest hindrance to the operation will be found in the ranks of the profession. There are always among us those who oppose anything radical in its nature, or, in fact, anything that is not antiquated. These will gradually become converted as did the German and French surgeons, who for a number of years derided the laparotomist as an adventurer and mad-man. It has become my practice to advise the operation as soon as the nature of the disease has been definitely decided upon. So far I have had no cause to regret having done so. All the cases upon which I have performed the operation—sixteen in number—being entirely relieved and capable of enjoying life as they did previous to becoming infected by the gonococci. The advantages which vaginal hysterectomy has over other modes of treatment are: (1) It rids the patient of the disease, which is not accomplished by any other mode of treatment. (2) If done in time, the ovaries are left in a healthy condition, thereby preserving for the patient those organs which have such a controlling influence over the nervous system. (3) In the removal of an offending organ the constant danger of the extention of the infection to the peritoneal cavity is likewise removed. (4) The patient's mental and physical condition improves rapidly. (5) The sexual instinct is left intact, and the possibility of its gratification remains normal—the patient thereby being saved the unhappy life that necessarily follows unsexing of the patient, which becomes necessary if the disease is allowed to pursue an uninterrupted course.

It might be argued that the operation is too serious for the purpose it is intended, or that it takes away from woman the possibility of becoming a mother. Statistics refutes the first. The death rate from vaginal hysterectomy—taking cases as they come—is scarcely 1 per cent., while at least 20 per cent. of the women affected with gonorrheal endometritis succumb to its sequelæ, and fully as great a per cent. of the remainder have their lives materially shortened by the debilitating influence of the disease upon their constitutions. The balance doubtless are the material out of which the doctors who condemn surgery make their living. As to the possibility of the patient becoming a mother, suffice it to say that she stands about as much show to become a mother after the operation as she did before, for, fortunately, the disease most always renders them sterile. I say fortunately for the sake of both mother and child. If pregnancy should take place a miscarriage will likely follow and the mother's life thereby be endangered. Should she by accident give birth to a child it will in all probability be affected with ophthalmia neonatorum and lose its sight, or at least have it impaired. Such women should not be allowed to have children even if they can.

The prevention of this disease should be given more attention by the profession in the future than it has been given in the past. It is a preventable disease, and therefore an unnecessary one. Its prevention can only be accomplished by education. On account of the nature of the disease and the inherent modesty of the human family such education has always been and probably always will be neglected. In the meantime such remedial measures as are at our disposal will have to be employed. Realizing the almost impossibility of a drug ever being discovered that will penetrate the endometrium and at the same time retain sufficient strength to destroy the gonococci, I shall continue in the future, as in the recent past, to advise that the offending organ be removed.

